# Case marking is different in monolingual and heritage Bosnian in digitally elicited oral texts

**DOI:** 10.3389/fpsyg.2022.832831

**Published:** 2023-01-09

**Authors:** Ilma Jažić, Natalia Gagarina, Alexandra Perovic

**Affiliations:** ^1^EMCL+, University of Groningen, Groningen, Netherlands; ^2^Leibniz-Zentrum Allgemeine Sprachwissenschaft, Berlin, Germany; ^3^Department of Linguistics, Psychology and Language Sciences, University College London, London, United Kingdom

**Keywords:** heritage language, bilingualism, case marking, narrative, nominal morphology, heritage grammars

## Abstract

Heritage languages may differ from baseline languages spoken in the home country, particularly in the domains of vocabulary, morphosyntax and phonology. The success of acquiring and maintaining a heritage language may depend on a range of factors, from the age of acquisition of the second language; quantity and quality of input and frequency of first language use, to non-linguistic factors, such as Socio-Economic Status (SES). To investigate case marking accuracy in heritage Bosnian in relation to these very factors, we recruited 20 heritage Bosnian speakers in Austria and Germany, and 20 monolingual Bosnian speakers in Bosnia, aged between 18 and 30 years. Participants were assessed remotely in two sessions, on a battery of tests that included a background language questionnaire investigating participants’ history of language acquisition, current usage and SES, and a newly adapted Bosnian version of the Multilingual Assessment Instrument for Narratives (MAIN). A significant difference in case marking accuracy was found between the two groups, despite the 97% correct performance in the heritage speakers, and an almost 100% performance of the monolinguals. In the heritage speakers group only, errors indicated a trend toward case system simplification as well as uncertainty in distinguishing between case meanings. The use of Bosnian, assessed through quantity and quality of input, as well as frequency of current usage, was shown to be a significant predictor of case marking accuracy in heritage speakers. In contrast, SES and age of acquisition of German did not play a role in these participants’ case accuracy. The observed patterns of quantitative and qualitative differences in the case marking accuracy between heritage Bosnian speakers and their monolingual counterparts, in the face of a high level of accuracy, contribute to our understanding of the heritage language attainment in more diverse language dyads where L1 is a lesser studied language.

## Introduction

Bosnian is a morphologically complex Slavic language which is relatively under-researched, thus belonging to the category of lesser-studied heritage languages (Scontras and Putnam, 2020). Currently, Bosnia boasts a population of 3.5 million citizens. With 1.5 million speakers abroad, a considerable number of Bosnian speakers use and learn Bosnian within a bi- and multilingual context. As a result of the war in the 1990s, many Bosnian-speaking families immigrated to German-speaking countries (Gamlen, 2019). They have continued using Bosnian as their home language, transferring it to their children who now speak it as a heritage language.

The focus of our study is inflectional case morphology of young adult heritage Bosnian speakers in a Germanophone context. Case is defined as overt marking of the syntactic or semantic relationship of the noun with other elements within the same clause or sentence (Velupillai, 2012). The case marking of a noun is typically realized through affixes. Case marking can also be exhibited on adjectives, pronouns and determiners, however, for the purpose of this study, the focus will be on noun case marking. In this study, we investigate case morphology marking in adult heritage speakers, compared to monolingual speakers. We also consider various linguistic and non-linguistic factors that have previously been found to influence accuracy of morphosyntax in heritage languages: L2 age of onset (Anderson, 1999; Gagarina and Klassert, 2018), input and usage of the heritage language (Gathercole and Thomas, 2009; Kupisch, 2019; Czapka et al., 2021), and Socio-Economic Status (SES) (Sánchez, 1983; Cobo-Lewis et al., 2002).

### Heritage bilingualism

The general consensus on the definition of a heritage language (HL) includes the following features: it is a minority language in a context of a majority language, HL speakers are bilingual and the majority language usually prevails as the dominant one in the adulthood (e.g., Lohndal et al., 2019). There are many aspects in which a HL may differ from the baseline/homeland language – the language as it is spoken in the home country: most notably in the domains of vocabulary, morphosyntax and pronunciation. In the domain of inflectional morphology, some cross-linguistic data point to a trend of rule simplification in the HL. Researchers argue that this language domain is particularly vulnerable to reanalysis of the underlying grammatical representation, a phenomenon also referred to as restructuring or, in some studies, variation (Montrul, 2015; Wiese et al., 2022). In terms of nominal morphology, this may be exhibited through a reduced or simplified case system, inconsistent use of gender, and subject–verb agreement errors. The simplification of the case system may also result in the omission of overt case markings (Montrul, 2015), thus resulting in a case system which reduces the opposition to nominative-accusative only in Russian, for instance (Polinsky, 2006, 2008). Other findings show oblique (non-nominative) cases in Russian heritage speaker production, however, their use is not always appropriate. For example, the loss of the differentiation between direction-location contrast, as expressed by the accusative and prepositional cases respectively (Isurin and Ivanova-Sullivan, 2008) as well as the use of nominative in the position of a direct object, the so-called *unification* of case features (Gagarina, 2017) has been observed.

On the other hand, a number of studies report contrasting results: that the heritage grammar shows no signs of simplification (Flores, 2015; Embick et al., 2020; Łyskawa and Nagy, 2020; Wiese et al., 2022). According to these authors, the variation found in the heritage grammar is a reflection of the variation that already occurs in the baseline grammar, differing only quantitatively – with heritage grammar having a higher incidence of variation. This discrepancy is attributed to the differences in the input received by monolingual and heritage speakers. The amount of language input available to heritage speakers is usually reduced compared to that of monolingual speakers (to be discussed below) and heritage speakers are more likely to receive input from the spoken register and/or non-standard variants. For instance, Łyskawa and Nagy (2020) found that case marking across heritage Polish, Ukrainian, and Russian was similar to that found in speakers of the languages spoken in these countries. Most variations observed in heritage languages were also noted in homeland languages (e.g., genitive-accusative substitution). The only exception was a default nominative assignment used solely in heritage languages. Case marking accuracy of heritage speakers has indeed been found to be robust, with the usual rates of accuracy reaching 90% and higher in different languages (Bolonyai, 2002; Hlavac, 2003; Rothweiler et al., 2010; Schmitt, 2010).

Child heritage speakers may demonstrate a slower rate of L1 case inflection acquisition compared to their monolingual counterparts. This involves a longer timeframe for developing case oppositions and uncertainty in determining the declension of nouns. Omitted and erroneous case marking forms are also observed in heritage speakers at an age when such a phenomenon is no longer found with monolingual children. Such a delay can occur if there is a considerable reduction in the amount of HL input upon L2 onset, as the case inflection may already be opaque and acquired relatively slowly even in a monolingual setting (Gagarina, 2011; Gagarina and Klassert, 2018). Additionally, some studies report a differential error pattern between structural and lexical cases in child heritage speakers. Structural case markings are more likely to be omitted, while lexical case markings show both omission and substitution errors (Bolonyai, 2002; Rothweiler et al., 2010).

### The role of linguistic and extralinguistic factors in heritage languages

Heritage speakers can either acquire the HL and the language of the environment simultaneously from birth or sequentially. In the latter case, the heritage speakers are raised in a monolingual HL environment until they enter the education system in the second language. This exposure usually occurs around the ages of 2 or 4, but it is not unusual for it to occur later, at the ages of 5 or 6. The age at which this exposure happens is referred to as the Age of Onset (AoO) or age of bilingualism (Kupisch, 2013). Distinctions are made not only between simultaneous and sequential bilinguals but also between different AoO groups within the sequential bilingual group. The reason these distinctions are made is because of the assumption that there exist multiple sensitive or critical age periods. Exposure to sufficient language input during these periods ensures a more successful acquisition of certain linguistic features. After these periods are complete, native-level attainment of those features within the first language timing and path is less likely. Informed by the findings on neurological development as well as the typical schedule of language acquisition, the proposed critical periods are ages 4–6 and ages 6–7 (Meisel, 2009, 2011). The onset of the L2 implies a decrease in the amount of HL input. This in turn may affect the level of success with which certain HL features are acquired or trigger attrition of already acquired HL features (Montrul, 2015).

The effect of the AoO of the L2 on the development and outcomes of heritage grammars has been widely investigated.^1^ It has been demonstrated in different linguistic domains, from phonology to morphosyntax (e.g., Flege et al., 1999). Some studies argue for a sequential bilingual advantage in HL over simultaneous bilinguals. This is ascribed to a longer HL monolingual period and a later AoO of the society language (SL). This effect of AoO was found in HL domains such as gender agreement (Anderson, 1999) and aspectual contrasts in Spanish (Montrul, 2002) as well as case inflection and expressive lexicon in Russian (Gagarina and Klassert, 2018). However, some domains of HL grammar fail to show an effect from AoO: definiteness in Turkish (Kupisch et al., 2016), sentential negation and wh-questions in Greek (Makrodimitris and Schulz, 2021) and verb inflections in Russian (Gagarina and Klassert, 2018).

Heritage language input and use are thus crucial in the investigations of HL development. Both are complex, multidimensional concepts which need to be carefully dissected. There are a multitude of possible sources of linguistic/HL input such as from family and peers, educational institutions (school, preschool, day care) as well as media (books, TV, music) (Unsworth, 2016). It is useful to consider both the quantity and quality of input and use (Kupisch, 2019). Quantity can be inferred from the number of people (parents, siblings, friends) speaking the HL, the number of visits to the country of HL and activities carried out in HL (Kupisch, 2019). Quality of HL input is commonly gauged by the linguistic richness of the input and contextual diversity of HL exposure. The HL may be spoken by individuals whose language is rich and of standard variety or has already undergone attrition; the HL can be exclusively spoken or also written; it can be exclusively informal or it can be provided in educational contexts (Kupisch, 2019; see also e.g., Unsworth, 2015, 2016).

The effect of HL input and use on the development of the HL in children has been shown to influence the speed and manner of acquisition across different linguistic domains such as vocabulary, morphosyntax and semantics (Thomas and Gathercole, 2005; Gathercole and Thomas, 2009; Paradis et al., 2011; La Morgia, 2015; Montrul, 2015; Unsworth, 2015, 2016; Gagarina and Klassert, 2018; Kupisch, 2019; Czapka et al., 2021; Makrodimitris and Schulz, 2021). Variation in the quantity and quality of input as discussed above is considered by some the fundamental determinant of the interindividual variation observed in bilingual language acquisition (Paradis, 2011).

The quantity of HL input is known to affect vocabulary size as well as diversity of produced morphemes: children receiving more input are reported to perform better than those with less input (Gathercole and Thomas, 2009; Unsworth, 2015, 2016; Czapka et al., 2021). Importantly, Gagarina and Klassert (2018) and Makrodimitris and Schulz (2021) report the use of the HL at home to be a significant predictor for the grammar domains under investigation in their respective studies. In their study of local and distant gender marking in Welsh with Welsh-English bilingual children, Thomas and Gathercole (2005) found the amount of input to influence speed of acquisition, especially with regards to the more complex and less transparent structures (such as possessive *ei* for masculine nouns in Welsh). Such structures are acquired later: a lower amount of HL input would not suffice in ensuring the successful attainment of the feature during the critical period for its acquisition. There is further evidence that the input received during childhood, as well as throughout life, is critical for the development and maintenance of the HL in adulthood. Another study by Gathercole and Thomas (2009) found that the vocabulary levels of adult heritage Welsh speakers were affected by both the input from their childhood as well as the consistency of input they received as adults (e.g., language of the partner). The amount of HL input and use is often related to the status that the language enjoys in the social environment of the heritage speaker. The social value attributed to the HL will determine whether the country’s policies allow for education in that language or how present the language is in the public sphere in general, all of which ultimately affects the success with which it is mastered (Montrul, 2015). In Gathercole and Thomas (2009), the authors are mindful of the fact that the Welsh-English community is quite stable and large, which is not usually the case for immigrant bilingual communities.

With regards to non-linguistic factors, the role of SES (most often measured *via* variables such as education, income, and occupational prestige) in language development in monolingual contexts is reported to be vital. A higher SES is known to correlate with more advanced lexical and grammatical skills (Hoff, 2006), where quantity and quality of language input, amongst other factors, is argued to be particularly relevant in early lexical development (Hart and Risley, 1995). As for heritage speakers, and especially adults, the relationship between HL development and SES is less well understood. Lower SES Spanish heritage speakers in the US were found to use more HL daily and achieve higher oral proficiency compared to their higher SES counterparts (Sánchez, 1983; Cobo-Lewis et al., 2002). On the other hand, in the study of Armon-Lotem et al. (2011) of Russian–Hebrew and Russian–German speakers, no effect of SES was reported on the L1 maintenance for the Russian–Hebrew cohort, but was present in the Russian–German cohort. The authors explain the lack of an SES effect in the Russian–Hebrew group by the SES homogeneity of that particular group.

In sum, while some factors such as AoO, the prestige of the home language, or SES have attracted more attention in the literature, the role of other factors, such as quantity, and especially quality of input and use in heritage languages, are less well researched and understood.

## Bosnian as a heritage language

### Basic characteristics of Bosnian

A south Slavic language, Bosnian shares many properties with other Slavic languages, such as rich morphology, relatively free word order and a lack of articles. Number, case and gender markings are fused into a single suffix and are marked on all nominal elements: nouns, pronouns, adjectives and some numerals. Additionally, all of the nominal elements within an noun phrase (NP) express number, case and gender agreement. Verbs can be inflected for person, tense, aspect and mood, while subject-verb agreement includes features of number, person, and gender.^2^ The sentence in the example (1) illustrates most of the characteristics above.

**Table:** 

(1)	Ona popravlja moju staru mašinu.
	She repair_3SG.PRS_ my_ACC.F.SG_ old_ACC.F.SG_
	machine_ACC.F.SG_
	“She is repairing my old machine.”

### Case morphology in Bosnian and its acquisition

The Bosnian case system differentiates between seven cases (see Table 1). Based on the class of the noun, there are three basic types of declensions. The first one consists of masculine (not ending in -a) and neuter nouns, the second contains nouns ending in -a (feminine and masculine), while the third declension solely accepts feminine nouns with a zero ending.^3^

**TABLE 1:** Cases in Bosnian and their prototypical function and meaning.

Case	Function	Meaning
Nominative	Subject	Labeling
Accusative	Direct object	Object/goal (with prepositions)
Genitive	Possessor, missing entity, genitivus partitivus	
Dative	Indirect object	Recipient/goal
Vocative	Addressing someone	
Instrumental	Device or company	Means and company
Locative	Prepositional phrase – verb complement	Topic and location

A pertinent phenomenon that occurs in case paradigms is syncretism – where distinct cases share the same form (see Table 2 for examples relevant to Bosnian noun declensions). The nominative case is syncretic with the accusative case in the paradigms for inanimate masculine nouns, all neuter nouns and feminine nouns with a zero ending. Therefore, in all of these paradigms, both the nominative case and the accusative case forms have a zero ending. For animate masculine nouns, the genitive case is syncretic with the accusative case. All paradigms also have syncretic forms for plural dative, locative and instrumental forms.

**TABLE 2:** Examples of Bosnian noun declension, for Masculine, Neuter and Feminine genders, singular and plural forms: “konj” horse; “dan” day; “selo” village; “ruka” hand; and “stvar” thing.

	Masculine		Neuter	Feminine	
	Animate	Inanimate		-A ending	Consonant ending
N sg	k``onj	dân	sèlo	rúka	stvâr
G sg	kònja	dâna	sèla	rúkē	stvâri
D sg	kònju	dânu	sèlu	rúci	stvâri
A sg	kònja	dân	sèlo	rúku	stvâr
V sg	k``onju	dâne	selo	rúko	stvâri
I sg	kònjem	dânom	sèlom	rúkōm	stvâri
L sg	kònju	dânu	sèlu	rúci	stvâri
N pl	kònji	dâni	sela	rûke	stvâri
G pl	kònjā	dánā	sèla	r``ukū	stvárī
D pl	kònjima	dânima	selima	rúkama	stvârima
A pl	kònje	dâne	sela	rúke	stvâri
V pl	k``onji	dâni	sela	rúke	stvâri
I pl	kònjima	dânima	selima	rúkama	stvârima
L pl	kònjima	dânima	selima	rúkama	stvârima

N, nominative; G, genitive; D, dative; A, accusative; V, vocative; I, instrumental; L, locative; sg, singular; pl, plural.

In order to better understand the properties of heritage Bosnian, here we provide a brief overview of monolingual child acquisition of case, in view of similarities between heritage speakers and child L1 learners (Polinsky and Scontras, 2020). The acquisition of nominal morphology and case in Bosnian children is not well documented, however, some evidence from Croatian does exist: the two languages are close enough to expect similar acquisition patterns in this domain of grammar. Before their second birthday, Croatian-speaking children already develop certain mini-paradigms (Kovačević et al., 2009). These paradigms are usually found for feminine nouns whose case forms are less syncretic. As such, they provide a clearer juxtaposition between the case markings in the input, which the children then utilize to construct mini-paradigms, usually contrasting 3–4 cases. All case markings emerge before age 1;10, with accusative markings first appearing at age 1;4, while locative and instrumental markings are among the last to occur at ages 1;9 and 1;10, respectively. At age 2;5, the distribution of cases already closely resembles that of the adult input language (Kovačević et al., 2009). The development of fully fledged paradigms for all lexemes in the child’s mental lexicon is, however, a long and complex process – case morphology is characterized by non-transparency and syncreticity cross-linguistically which influence the rate at which it is acquired (Xanthos et al., 2011). Incorrect case forms of certain nouns can be found in pre-school as well as school age (Kovačević et al., 2009; Vrsaljko and Paleka, 2018). A common error of using the locative^4^ (used to signify location) instead of accusative case (used to signify direction) appears in 2-year-olds: “i onda smo išli na placu [*]” (Hržica and Peretić, 2015), and is seen even later, at age 6: “…dok je on išao u krevetu [*]” (Hržica and Lice, 2013). If case poses a challenge for monolingual L1 acquisition, it can serve as a valuable foundation for making predictions on the outcomes of heritage language acquisition (Polinsky, 2018; Polinsky and Scontras, 2020). We could thus expect heritage speakers to diverge from standard usage of Bosnian with case markers that seem most problematic for Croatian child language L1 speakers, e.g., accusative with nouns signifying direction.

### Heritage Bosnian

There are a handful of studies on heritage Bosnian, though mostly in an English-speaking context and amalgamated with the closely related Croatian and Serbian into heritage BCMS (Bosnian, Croatian, Montenegrin, Serbian) studies. While the majority of these studies take on sociolinguistic issues, such as quality of education in the heritage language or attitudes toward the HL (e.g., Ćatibušić, 2019), Hlavac (2003) focuses on the morphology of heritage Croatian speakers. This study included 100 participants aged 16–32 who were either born in Australia or moved there before the age of 5 with parents who originated from either Croatia or Bosnia and Herzegovina. The corpus created consisted of 15–20 min of transcribed speech segments of answers to open-ended questions as well as descriptions of visual stimuli. Some information on linguistic background, such as order of acquisition and use of HL with friends and family were gathered through a structured questionnaire, but no information on the level of education or other SES factors were provided. The recorded background linguistic factors were not included into the analyses as potential explanatory variables.

Heritage speakers used target case marking in more than 90% of cases. An example of non-target case marking is given in (2): the noun *rodbina* “extended family” is used erroneously in the unmarked nominative form instead of the overtly marked accusative form *rodbinu.* In their examples of intra-word code-switching, participants sometimes used an appropriate Bosnian case marker on the English NP (example 3). In some instances, however, an NP contained an unintegrated, directly transferred English noun as its head (example 4). In such cases, the rest of the NP constituents which are congruent with the head noun, such as attributives and determiners, had a higher incidence of non-target markings. Thus in example 4, the preposition *na* “on” requires the locative case, but due to the unintegrated noun “side,” the dependent attributive “other” is in the unmarked nominative case. The example in (5) illustrates the case and number mismatch found in heritage Croatian NPs: the possessive *njegov* “his” is singular and nominative, while the head *prijatelje* “friends” is plural and accusative.

**Table:** 

(2)	… I	tu	imamo rodbina*
	and	here	have_1PL.PRS_
	extended family_NOM.F.SG_		
	…“and here we have extended family”		

**Table:** 

(3)	… I tamo sam	dobio	posao
	and there be_1PSG.AUX_	got_3SG.M.PTCP_	job_ACC.M.SG_
	u hospital-u za treću godinu*		
	in hospital_LOC.M.SG_ for third year_GEN.F.SG_		
	… “and there I got a job in a hospital for a third year”		

**Table:** 

(4).	… Gdje	je	plaža,
	Where	be_3PSG_	beach
	na	drugi	*side* ima …*
	on_(+LOC)_	other_NOM.M.SG_	side have
	“…where the beach is, on the other side there is…”		

**Table:** 

(5)	Trebam	vodit	moj
	Must_1SG.PRS_	take_INF_	my_NOM.M.SG._
	brat	i njegov	prijatelje*
	brother_NOM.M.SG_	and his_NOM.M.SG._	friend_ACC.M.PL_
	“I must take my brother and his friends”		

Research on heritage BCMS in the German-speaking context have also had a sociolinguistic focus, especially on the issue of cultural identity (Savić, 1989; Schlund, 2006; Randjelovic, 2019). The main insights into the language features of heritage BCMS speakers in a Germanophone context comes from studies by Hansen et al. (2013) and Hansen (2018)^5^. These authors investigated heritage BCMS speakers aged between 20 and 32. Participants were either born in Germany or moved there before the age of five from either Bosnia and Herzegovina, Croatia, or Serbia. The corpus consisted of qualitative interviews in heritage BCMS from 11 participants, supported by written production data (essays written in a heritage language class) and speech samples elicited on the basis of four pictures. In line with Hlavac (2003), case incongruity between head nouns and their dependents was also observed (example 6). These BCMS heritage speakers seem to pattern with American Russian heritage speakers regarding the difficulty observed in dealing with the two-way prepositions assigning either accusative or locative (Isurin and Ivanova-Sullivan, 2008). In example (7) both cases are used, incurring another instance of case mismatch within an NP.

**Table:** 

(6)	… I	kod nas	su	
	and	at	we_GEN_	be_3PL.PRS_
	one	turski	krovove*	
	this_ACC.M.PL_	Turkish_NOM.M.PL_	roof_ACC.M.PL_	
	“And we have those Turkish roofs”			

**Table:** 

(7)	Onda kaže	na jednoj	ruku
	then say_3SG.PRS_	on one_LOC.F.SG_	hand_ACC.F.SG_
	isto	što	nisam
	same	what	_NEG_._1SG.AUX_
	bio	uvek	
	be_3SG.M.PST_	always	
	“Then she says the same on the one hand that I was never there.”		

There are several instances that indicate transfer of a German argument structure, resulting in incorrect case marking. In example (8), the heritage speaker uses the preposition *protiv* “against” with an accusative, which is a structure corresponding to the German counterpart “gegen” but is erroneous as the BCMS preposition assigns the genitive. Similarly, in the heritage BCMS sentence (9), the existential verb *ima* “have” assigns the accusative, as is the norm in German, but this takes the nominative case in BCMS^6^. Heritage speakers in this corpus also exhibited deviations in gender agreement between nouns and their determiners, as observed in example (10).

**Table:** 

(8)	i dobili	jednu	jednu
	and get_1PL.M.PTCP_	one_ACC.F.SG_	one_ACC.F.SG_
	utakmicu	i… protiv	
	match_ACC.F.SG_	and … against(+ GEN)	
	Mađare*	dva-dva	odigrali
	Hungarian_ACC.PL_	2:2	play_1PL.M.PTCP_
	“We had one match against the Hungarians, we played 2:2.”		

**Table:** 

(9)	tamo u. ima	njemačku
	There at have_3SG.PRS_	German_ACC.F.SG_
	poštu	ili Telekom u Zagrebu*
	post-office_ACC.F.SG_	or Telekom in Zagreb_LOC.SG_
	“There at. in Zagreb there is a German post office or Telekom.”	

**Table:** 

(10)	i na taj	vreme	šta
	and on this_ACC.M.SG_	time_ACC.N.SG_	what
	da	uspijem?*	
	COMP	manage_1SG.PRS_	
	“What can I manage in this time?”		

Based on the studies above, the general features of heritage Bosnian have been documented and outlined. Building on this foundation, this study aims to provide a more precise picture of heritage Bosnian nominal morphology and to explain its variation.

## The present study

The current study investigates case marking in adult heritage Bosnian speakers having grown up with German as their societal language, compared to adult Bosnian monolinguals. The first research question asks whether case marking accuracy differs between heritage speakers and monolinguals in obligatory contexts in elicited narratives. Based on previous findings from heritage Slavic languages, we predict, (a) lower accuracy in case marking in heritage Bosnian compared to the monolingual/baseline language; and (b) a restructuring of the heritage case system. By restructuring we mean the omission of overt case markings and reduction of case oppositions.

Our second research question is concerned with factors that influence the accuracy of case marking in the heritage speakers. Previous studies have emphasized the role of both linguistic (e.g., use and input) and extralinguistic factors (e.g., SES) in the development and maintenance of language proficiency as well as discrete features of grammar in children (Armon-Lotem et al., 2011; Gagarina and Klassert, 2018; Makrodimitris and Schulz, 2021). It is not well established whether these effects persist into adulthood for heritage speakers. We therefore ask whether the usage of heritage Bosnian, age of German onset and participants’ SES predict case marking accuracy in narratives of adult Bosnian heritage speakers.

## Materials and methods

### Participants

We recruited two groups of participants aged between 18 and 30: 20 adult Bosnian heritage speakers (15 female) from Germany and Austria and a control group of 20 adult native Bosnian speakers (11 female) from Bosnia (see Table 3). All of the participants were healthy adults, with no neurological conditions and normal or corrected-to-normal vision. The heritage speakers had at least one Bosnian parent. They grew up in a German-speaking country or moved to one before the age of four. All of the participants in the control group were monolingual speakers who were born and had lived all of their lives in Bosnia and Herzegovina. They came from monolingual households, though a majority of them learnt at least one foreign language during their primary and secondary education. None had a university-level degree in a foreign language.

**TABLE 3:** Participant information.

Group	*N*	Age – Years (*SD*)	Years of education (*SD*)	Age of L2 onset (*SD*)
Monolingual	20 (11 females)	24.05 (3.06)	15.3 (2.36)	
Heritage speakers	20 (15 females)	23.35 (2.99)	14.92 (2.62)	3.8 (1.6)

### Background measures

Information about the participants’ history of language acquisition and current usage was collected through a Language Background questionnaire, adapted to Bosnian from Lloyd-Smith (2020). Using the information collected through the questionnaire and adapting the procedure outlined in Lloyd-Smith (2020), a Bosnian Use Score for heritage speakers was calculated. This score quantifies heritage language use by considering four core aspects: Language Use at Home, Quality of Language Use, Current Language Use and Time Spent in Heritage Country. A detailed explanation of the weighted score calculation can be found in Table 4. The maximum possible score is 28.5, and for the current group of participants the mean score was 17.56 (*SD* = 3.03).

**TABLE 4:** Bosnian usage score calculations.

Types of use	Scoring
**Language use at home**	
L of father L with father L of mother L with mother L siblings L grandparent L partner Distant relatives L at home (before age 6) L at home (after age 6)	1 pt = Bosnian 0.5 pts = Bosnian and German 0 pts = German
**Quality of language use**	
Number of years schooling in Bosnian	6 + years = 3 pts; 3 + years = 2 pts; 1–2.9 years = 1 pt; 0 year = 0 pts
Bosnian studies at the University	Yes = 1 pt; No = 0 pts
Number of Bosnian University courses	7–9 = 1.5 pts; 4–6 = 1 pt; 1–3 = 0.5; 0 = 0 pts
Number of contact types with Bosnian	Listening/speaking/reading/writing = 3 pts; 1 of 4 missing = 2 pts; 2 of 4 missing = 1 pt
Long period of Bosnian non-use	No = 1pt; Yes = 0 pt
**Current Language Use**	
Relative use of Bosnian vs. German	Bosnian 100% = 3 pts; 75% = 2.5 pts; 50% = 2 pts; 25% = 1 pt; 0% = 0 pts
L at work/school	1 pt = Bosnian 0.5 pts = Bosnian and German 0 pts = German
L at home	
L with friends	
**Time spent in heritage country**	
Number of years spent in Bosnia	2.1 + years = 3 pts; 1.1–2 years = 2.5 pts; 6.5–12 months = 2 pts; 4–6 months = 1.5 pts; 3–4 months = 1 pt; 1–2 months = 0.5 pts
Number of visits in past 5 years	7 + = 2 pts; 4–6 = 1.5 pts; 1–3 OR every other year = 1 pt; 0 = 0 pts

Additional background measures included Years of Education, as a measure of SES, and AoO for German. For the monolingual group, the mean number of years of education was 15.3 (*SD* = 2.36), not statistically significantly different (*p* = 0.637) from that of the heritage speaker group, whose mean number of years of education was 14.92 (*SD* = 2.62). The mean AoO of German for the heritage group was 3.8 (*SD* = 1.60).

### Procedure and scoring

The data collection took place over the course of the summer of 2021. The test battery consisted of the aforementioned Language Background Questionnaire and the newly adapted Bosnian version of the Multilingual Assessment Instrument for Narratives (MAIN) (Gagarina et al., 2012, 2019b). The data was collected remotely using the conferencing software Zoom.

For the heritage speakers, there were two sessions per participant, lasting roughly 15 min each. One session consisted of filling out the Language Background Questionnaire followed by a MAIN assessment in Bosnian administered by the first author. The second session consisted of a MAIN assessment in German administered by a student research assistant working for the ZAS, Berlin, Germany. Monolingual participants were administered the MAIN and the Language Background Questionnaire in the course of one session. The counterbalancing for language and story order followed the procedures outlined in the MAIN manual (Gagarina et al., 2012, 2019a).

The procedure of the MAIN administration in Bosnian included the elicitation of two stories, “Cat” and “Baby Birds,” through the telling mode. The order of the stories was counterbalanced with half of the participants telling the Cat story first, while the other half told the Baby Birds story first. The MAIN was adapted for online administration closely following the offline version of the instrument. In the offline administration of the assessment, the participant chooses one of three envelopes presented and then proceeds to take out the story picture strip from that envelope. This way the investigator supposedly cannot know which story the participant will choose nor can the investigator see the pictures while the participant is describing them. However, in the online version the investigator is required to share their screen with the PowerPoint slides containing the picture sequences, which makes it impossible to maintain the pretense of the investigator not knowing which story was chosen and what the pictures look like. The instructions were thus modified to include the line: “While you are talking, imagine that I cannot see the pictures.” Similarly, in the online version, it is the investigator who controls the progression of the picture sequence, unlike in the in-person administration. The initial slide shows three envelopes, from which the participants need to choose. The following slide displays an embedded video with the full story unfolding, one picture at a time. Three slides each containing a picture pair were then shown, mimicking the unfolding of two pictures at the time. For the comprehension part of the test, a slide showing all six pictures, was displayed with a red frame around the pictures relevant to the question being asked.

For the purposes of the current analyses, the accuracy of all six case markings (nominative, genitive, dative, accusative, instrumental and locative) in obligatory contexts was scored and examined only on nouns (case inflection on adjectives and other lexical items were not included in the analysis). An item for analysis equaled a case inflection on a noun. Each token rather than type of noun case inflection produced within a participant’s narrative was considered an item. Items were scored 1 for accurate – on target inflection, or 0 for inaccurate – non-target inflection (encompassing omission, substitution and novel marking). The inflection accuracy per case was also analyzed.

## Results

As there was no significant difference in the scores between the stories, the case accuracy scores were merged for analysis (Table 5).

**TABLE 5:** Case marking raw scores and percentages per condition and story.

	Heritage speakers		Monolinguals	
	Cat	Baby Birds	Cat	Baby Birds
Nominative	99.2% (2/279)	97.5% (8/320)	100% (212)	99.4 (1/196)
Genitive	98.9% (1/94)	100% (66)	100% (69)	98.6 (1/72)
Dative	100% (9)	92.8% (1/14)	100% (11)	100% (9)
Accusative	93.8% (19/309)	96.4% (7/199)	99.5% (1/248)	100% (168)
Instrumental	93.4% (3/46)	96.6% (1/30)	100% (31)	100% (23%)
Locative	97.4% (2/79)	97% (2/70)	100% (72)	100% (66)
Total score	96.7% (27/814)	97.3% (19/700)*	99.8% (1/643)	99.6% (2/534)
*P*-value	0.496		0.459	

*Total score including a single correct instance of the vocative case.

Motivated by the first research question, a logistic mixed effect model was fitted using the lme4 package in R (Bates et al., 2015). This model predicted accuracy based on the group (monolingual or heritage speaker) while allowing varying intercepts for subjects and items. The results from this model are found in Table 6. Group was shown to be a significant predictor of accuracy, β = 2.66 (*SE* = 0.72), *z* = 3.67, *p* = 0.0001.

**TABLE 6:** Regression coefficients of group on case marking accuracy.

		Fixed effects			Random effects	
					By participant	By item

**Parameters**	**Estimate**	***SE***	**Wald *z***	***p***	***SD***	***SD***
(Intercept)	3.82	0.36	10.53	<0.001	1.10	0.16
Group	2.66	0.72	3.67	<0.001		
Observations	2691					
Model equation	Accuracy ∼ Group + (1 | ID) + (1 | Trial)					

**FIGURE 1:**
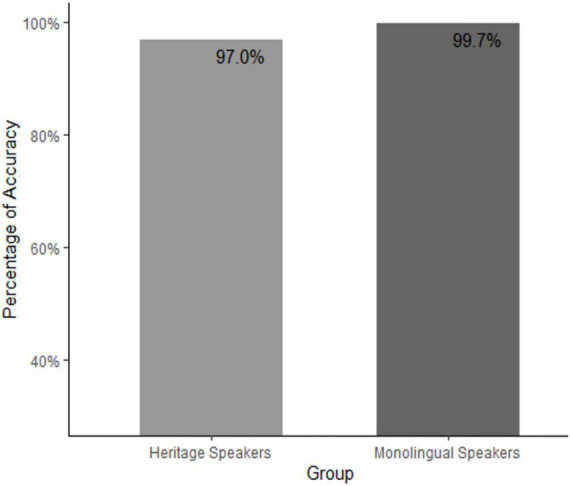
Percentage of case marking accuracy per group.

Looking at the accuracy rates between the two groups based on raw data, it is possible to deduce the direction of this difference (see Figure 1). Monolingual speakers had an almost perfect performance (99.7%, 3/1174), while the heritage speakers had a slightly lower accuracy rate (97.0%, 46/1465). Using the emmeans package in R, the odds ratio of the two groups was calculated (OR = 0.0698, 95% CI:0.016,0.289) (Lenth et al., 2021). With the help of the R package effect size and applying the “Chen, 2010” rule, the effect size was estimated to be very small (Chen et al., 2010; Ben-Shachar et al., 2020).

The second research question focused on the heritage speaker group. In order to investigate whether accuracy in the case marking of heritage speakers can be predicted by their usage of Bosnian, quantified through the Bosnian Usage Score (BUS), another logistic mixed effect model with random intercepts for participants was fitted (see Table 7). AoO and SES were added to the model as additional predictors. The model showed that the only significant predictor of accuracy was the BUS, with a higher score increasing the probability of higher case marking accuracy, β = 0.30 (*SE* = 0.09), *z* = 3.20, *p* ≤ 0.001.

**TABLE 7:** Regression coefficients of Bosnian Usage Score (BUS) on case marking accuracy of heritage speaker.

		Fixed effects			Random effects
					By participant

**Parameters**	**Estimate**	***SE***	**Wald *z***	***p***	***SD***
(Intercept)	–2.96	1.98	– 1.49	0.13	0.73
BUS	0.30	0.09	3.20	<0.001	
Years of Education	0.13	0.09	1.33	0.18	
Age of Onset	–0.10	0.16	–0.63	0.52	
Observations	1514				
Model equation	Accuracy ∼ BUS + Years of Education + Age of Onset + (1 | ID)				

### Case marking error analysis

Both omission and substitution errors were observed in the case marking of heritage speakers. There was one instance of novel marking – the case suffix did not correspond to any existing case marking suffixes. In some cases of omission, it was impossible to tell whether the error was a genuine case marking omission or a substitution with the nominative case, which takes a null ending. In other cases, this differentiation was possible: (1) when the NP contained other elements such as adjectives, demonstratives, etc. which exhibited congruency and were overtly inflected in the nominative; (2) with nouns belonging to paradigms in which the nominative form is overtly marked. However, having no means of verifying the substitution claim in other instances, all forms with zero marking in contexts requiring overtly inflected, non-nominative case, were considered omissions (example 11).

**Table:** 

(11)	Mačka	skače	na leptir-Ø*
	Cat_NOM.F.SG_	jump_3SG.PRS_	on butterfly- Ø
	“The cat is jumping on the butterfly”		

Within the substitution errors, there emerged two specific subgroups of errors. The first subgroup occurred when the case assigners were the so-called “two-way” prepositions. These prepositions can assign either accusative or locative/instrumental case, depending on whether they express directionality (accusative) or location (locative/instrumental). Our heritage speakers were found to misselect the appropriate case marking, using locative instead of accusative and vice versa (examples 12 and 13 respectively).

**Table:** 

(12)	…Pala	njegova lopt-a
	fell3PSG.PST.PERF	his ball-NOM.SG.F
	u jezer-u*	
	in lake-LOC.SG.N	
	…“His ball fell in the lake”	

**Table:** 

(13)	…Vidi	rib-e
	see3PSG.PRS.PERF	fish-ACC.SG.F
	u kant-u*	
	in bucket-ACC.SG.F	
	…“Sees the fish in the bucket”	

The second subgroup involved the substitution of the accusative case with the nominative case and vice-versa. The former type of substitution was more prevalent than the latter (9/15). In these instances, speakers assigned the nominative case to a direct object, and as noted above, this was apparent through the case agreement of the other NP elements or the overtly marked nominative form of the noun (example 14).

**Table:** 

(14)	…Jedn-a	mac-a
	one- NOM.SG.F	cat-NOM.SG.F
	koj-a	je
	who-F	beAUX.PRS.3SG
	ugleda-la	lijep-i
	see-PST.PTCP.F	beautiful- NOM.SG.MASC
	žut-i	leptir-Ø*
	yellow- NOM.SG.MASC	butterfly- NOM.SG.MASC
	…“A cat who saw a beautiful yellow butterfly”	

## Discussion

In the first study to focus on case marking in heritage speakers of a lesser-studied language, Bosnian, our HL participants showed an exceptionally high overall case marking accuracy, at 97% correct. This result is in line with previous findings (Bolonyai, 2002; Hlavac, 2003; Rothweiler et al., 2010; Schmitt, 2010). Our monolingual participants, Bosnian speakers from Bosnia, performed at ceiling. Group was a significant predictor of case marking accuracy, therefore our initial prediction of lower accuracy of case marking in heritage speakers was confirmed. However, the effect size was small, so caution should be exercised when interpreting the magnitude of this difference. The second prediction concerned the nature of case marking in heritage languages, namely the restructuring of the heritage case system through omission of overt case markings and reduction of case oppositions. Both these types of phenomena were observed in the current data.

### Error types

#### Nominative-accusative substitution

In line with previous studies, our heritage speakers produced omission and substitution errors (Bolonyai, 2002; Polinsky, 2006; Rothweiler et al., 2010; Gagarina, 2011; Montrul, 2015). The omission error occurred mostly with the direct object, which was supposed to carry an overt accusative marking. In all instances of omission of the direct object case, the null marked form corresponded to the nominative form. When categorizing these errors, the more conservative estimate that these were omission errors was used, however, one could also argue that these were instances of nominative-accusative substitution, at least for the first declension feminine and accusative nouns. Such an interpretation perhaps holds some merit. In all cases of overt substitution of accusative with nominative, it was the direct object which erroneously took the nominative marking. This could be interpreted as a trend toward the leveling of nominative and accusative cases, where direct objects are assigned the nominative case, similarly to heritage Russian. This case system reduction could be motivated by the overwhelming presence of nominative-accusative syncretism in some BCMS noun declension paradigms. While the spreading of syncretism has been argued to underlie the changes in case marking observed in Heritage Slavic speakers by Łyskawa and Nagy (2020), the number of errors produced by our participants is too small and does not cover nouns from different declensions for us to make any firm conclusions.^7^

#### Two-way preposition case assignment

As previously observed in adult and child heritage Russian speakers and adult heritage BCMS speakers, Bosnian heritage speakers in the current study also exhibited some difficulty with two way prepositions and accusative and locative alternation (for BCMS: Hansen et al., 2013; Hansen, 2018; for Russian: Isurin and Ivanova-Sullivan, 2008; Schwartz and Minkov, 2014). The accusative and locative were used interchangeably and indiscriminately with the two-way prepositions. This indicates a disregard for the accusative-locative distinction maintained by whether the preposition assigns the meaning of direction or location respectively. The two-way preposition expressing the same distinction with an accusative-dative alternation also exists in German, suggesting that this lack of discrimination is not influenced by the dominant language. Isurin and Ivanova-Sullivan (2008) suggest that such errors emerge due to the “reanalysis of case functions such as direction, location, means” (p. 81). There does seem to be some consistency in the way these errors were made by our participants, with substitutions involving two-way prepositions rarely implicating those cases not assigned by the preposition.

One interpretation may be that there exists a productive rule in the heritage grammar in which two-way prepositions assign their respective cases, but the distinction between the meaning of the cases is unclear to the speakers, due to reanalysis. Tentatively, it could be suggested that the relative lateness in locative emergence as documented for monolingual Croatian children and bilingual Russian-German children does not allow for a long enough rehearsal period (Kovačević et al., 2009; Gagarina, 2011). Note that this is one of the least frequently used cases in BCMS, both in adult input and child usage (Lukatela et al., 1980; Kovačević et al., 2009), and prone to errors in children as old as 6 (Hržica and Lice, 2013; Hržica and Peretić, 2015). Looking into the interaction of lateness in the locative emergence and the amount of input available for heritage speakers to form rules regarding two-way preposition usage could be the first step in solving this puzzle (Schwartz and Minkov, 2014). This suggestion is also brought forth by Klinge (2010) who observed a higher frequency of “divergent uses” (p. 144) for German two-way prepositions compared to one-way prepositions by German-French bilingual children. As Polinsky (2006) suggested, another possibility could be that heritage speakers retain “chunks” without having a productive rule in place which would allow for generalization. In this case a chunk would consist of a preposition and a noun (either in accusative or locative) which has been memorized from the input and is utilized at random without a productive rule which would determine the correct selection of the case based on the direction/location distinction. Future studies could further investigate this explanation *via* more constrained tasks.

#### Within noun phrase case mismatch

Instances of case mismatch within the NP were observed in our sample of Bosnian heritage speakers, in line with findings from both Hlavac (2003), Hansen et al. (2013) and Hansen (2018). In the example (15), we can see an instance of both case and number mismatch occurring within a single NP. This could potentially be construed as a transfer from German in which case marking is overt on articles, adjectives and pronouns, while the nouns remain largely unmarked (Blake, 2001).

**Table:** 

(15)	…Jedna	ptica	sa
	One_NOM.F.SG._	bird_NOM.F.SG._	with
	svojom	bebe*
	her.own_INS.F.SG_	baby_NOM.F.PL_
	…“A bird with her babies”		

#### Unintegrated noun phrase heads

Hlavac (2003) noted instances of both English-origin nouns which were integrated into a BCMS clause by taking on overt markings of BCMS (e.g., u hospital-u) as well as English-origin nouns which were unintegrated and simply inserted into the BCMS clause. In the present data only unintegrated German nouns were found. These unintegrated head nouns had a similar effect on the other congruent NP elements to the one observed by Hlavac: the quantifier in (16) and the deictic pronoun in (17) are both used with an unmarked case. Since the unintegrated head noun that governs the other NP elements lacks the appropriate language specific features such as gender and case, the agreement is unable to be checked and the dependents appear in unmarked forms.

**Table:** 

(16)	…Na prvoj	slici	ima
	On first_LOC.F.SG._	picture_LOC.F.SG_	have_3SG_
	jedan	baum
	one_NOM.M.SG_	baum
	…“On the first picture there is a tree (baum)”		

**Table:** 

(17)	I	ona	hoće	da
	And	she	want_3SG.PRS_	COMP
	ganja	to	schmetterling*	
	chase_INF_	that_NOM.N.SG._	schmetterling	
	“And she wants to chase that butterfly (schmetterling)”			

This pattern is far from unique to heritage BCMS: see e.g., Putnam et al. (2021) for discussion of similar examples from other HLs, and possible explanations.

### Other errors related to case

Patterns observed in some of our participants can be connected to case morphology in a more implicit manner. The example (18) shows an error in gender assignment: the speaker mistakenly assigns the feminine gender to the neuter noun *gnijezdo* “nest.” There are two indicators of this error: (1) the reflexive possessive preceding the noun is feminine; (2) the incorrect locative form of the noun corresponds to the locative form found in the paradigm of feminine nouns ending in -a. These two mistakes jointly suggest that the speaker believes that citation form of this noun is something similar to gnijezda*. She still maintains the gender agreement within the NP and assigns the correct case and number to the noun, but due to the erroneous gender assignment, an incompatible declension paradigm is applied resulting in a distinctly invalid noun form. Case and gender are related categories, thus errors in gender marking might reveal deeper understanding of the processes of heritage changes in the nominal morphology, in general, and case, in particular.

**Table:** 

(18)	…sjedile	su
	…sit_3PL.PTCP_	be_AUX_._3PL_
	male	ptičice
	little_NOM.F.PL_	bird_NOM.F.PL_
	u svojoj	gnijezdi*
	in their-own_LOC.SG.F_	nest_LOC.SG.F_
	“little birds were sitting in their nest”	

### General discussion

As evident in the examples above, there is a systematicity to the errors observed in the data across our participants. These could indicate a systematic restructuring of the underlying heritage grammar. Patterns of case leveling and substitutions similar to the ones found here have been observed in heritage speakers of the related heritage Russian (Polinsky, 2006, 2008; Isurin and Ivanova-Sullivan, 2008). These cross-linguistic findings lend credibility to the argument of heritage grammar restructuring (Putnam et al., 2021). However, we would certainly be remiss not to take into account the possibility that these patterns actually originate as a variation found in the homeland grammars, but are exacerbated by the specific conditions of heritage language acquisition (Flores, 2015; Bousquette and Putnam, 2020; Łyskawa and Nagy, 2020; Wiese et al., 2022). For example, the interchangeable use of accusative and locative with the two way prepositions observed in our data could be associated with the non-standard varieties of BCMS. In some dialects spoken in Serbia and Montenegro, accusative rather than the normative locative/instrumental is consistently used (Ivić, 1985). Thus the locative in “ja sam u trećem razredu” (I am in the third grade) in the standard form of BCMS can vary with the accusative: “ja sam u treći razred,” while the instrumental denoting place in the PP “pod slamom” varies with the accusative “pod slamu” (under the hay) (Ivić, 1985, p. 104). While the locative or instrumental denoting place are not used in these dialects, it is possible that speakers of such dialects occasionally use them, especially when in contact with speakers of the standard form, as they do not have the grammatical representation of the locative/instrumental (we thank Boban Arsenijević for this insight). This may result in hypercorrection, with the result of locative (or instrumental) being used in the environments where accusative should be used. Such a pattern has been informally observed by the third author in Albanian-Montenegrin bilinguals living in Montenegro: “Išao sam u Podgorici” (I went to Podgorica). Unfortunately, we are missing key information in order to make any sound judgment of this argument. There is no large corpora of spoken BCMS and their respective dialects to give us an insight into the possible variations currently present in the homeland variants. Our own sample of homeland speakers was fairly homogeneous: almost all of them came from central Bosnia, while our heritage speakers may have been exposed to a more diverse input as they are more likely to be surrounded by speakers from different areas within the BCMS dialect continuum spoken by the diaspora communities from former Yugoslavia. However, our questionnaire did not encompass questions on possible dialectal variations that our participants were in contact with. Such an analysis will hopefully be possible in the future, as there is a great need (both for clinical and research purposes) for a corpus representative of the diversity present in BCMS dialects, especially in the spoken register.

### The role of linguistics and non-linguistics factors: Language use, age of L2 onset and socio-economic status

Our measure of language use, the Bosnian Usage Score (BUS), was shown to be a significant predictor of case accuracy. These results are consistent with the previous research, which has demonstrated that HL input and use affect the development and maintenance of its inflectional morphology (Gathercole and Thomas, 2009; Putnam and Sánchez, 2013; Unsworth, 2015, 2016). Neither the AoO of L2 German nor the SES status as conveyed through the number of years of education were significant predictors of Bosnian case marking accuracy.

The lack of an AoO effect is not an isolated result, as AoO has not always been found to predict inflectional morphology accuracy in heritage speakers (Kupisch et al., 2016; Makrodimitris and Schulz, 2021). Our findings regarding case marking are in direct contrast to those of Gagarina and Klassert (2018), which showed AoO to be a significant predictor of case marking in heritage Russian. However, this discrepancy could be explained by a crucial difference between the populations: in contrast to our adult heritage speakers, Gagarina and Klassert’s participants included very young children, aged 26–98 months. The acquisition of case morphology in monolingual BCMS speaking children is a long process with errors still being registered at preschool age, at 5–6 years old (Vrsaljko and Paleka, 2018). It is thus possible that long-term continuous input and use exert a greater influence over the adult HL grammar outcomes, outweighing the effect of the L2 AoO. The fact that both studies found use at home (this factor constituting a large portion of the BUS) a significant predictor of case marking accuracy, lends credibility to this proposal. The lack of SES effect on heritage Bosnian case marking accuracy cannot be explained through the homogeneity of the group as in Armon-Lotem et al. (2011). Our group of heritage speakers was fairly heterogeneous in terms of their education, with the years of education ranging from 10 to 19. A more comprehensive SES score including additional SES variables such as the participant’s income bracket or the level of parents’ education may have constituted a better proxy for the SES as a whole and showed different results. Note also that in her study of the relationship between SES and proficiency scores in bilingual children, De Cat (2020) found that SES advantage only existed in cases of considerable, above-average amount of exposure. The current sample size is too small to perform a separate and reliable analysis for the participants with an above-average BUS and test whether our data would support this finding as well. It is also worth bearing in mind that SES may affect language outcomes in children and adults differently: both Armon-Lotem et al. (2011) and De Cat (2020) focus on children, making their results less applicable to our investigation of HL in adults.

## Summary and conclusion

The current study contributes to a growing body of research mapping out the characteristics of heritage BCMS, relying on the online administration of a narrative task. No study so far has examined structured narratives of heritage Bosnian, nor focused specifically on its nominal morphology. Whereas all of the heritage Bosnian studies previously conducted were of a qualitative nature, this study additionally provides quantitative evidence for divergent outcomes in heritage Bosnian grammar. The meticulously controlled tools and methods used to compile the corpus presented here allow for replicability lacking in earlier research. Moreover, prior studies either did not take into account linguistic background (AoO, HL use and input) (Raecke, 2006; Hansen et al., 2013; Hansen, 2018) or, if they did, no attempts were made to relate these factors systematically to the linguistic phenomena observed in the heritage language (Hlavac, 2003). The current study not only gathered relevant data on a range of linguistic factors such as AoO, HL input and usage and non-linguistic factors such as SES across participants, but this information was also utilized to quantitatively evaluate which of these factors are crucial to the development of specific HL domains. From a broader perspective, the results of the study enrich our understanding of the language change and add knowledge on the directions of HL restructuring.

## Limitations and future directions

One issue to be considered is the appropriateness of performing a comparison between adult heritage speakers and their monolingual age-matched counterparts in the homeland. According to Polinsky (2018) this comparison might not be particularly useful due to the difference in the input received, especially in adolescence and adulthood. The monolingual environment allows for the development of language novelties that may be unattainable for the heritage speaker group due to the difference in the amount and quality of input. This argument is legitimate, and perhaps an even more informative profile of adult Bosnian heritage speakers could be drawn up by simultaneously investigating the language of monolingual Bosnian children, child heritage speakers or first-generation immigrants. Examining the development of Bosnian in both monolingual Bosnian children and their heritage speaker counterparts of various age groups could provide a clearer picture of the trajectory of language acquisition which leads to the outcomes witnessed in the current data. The same applies for the language of first-generation immigrants, whose input helped shape the language of the heritage speakers. However, limited resources prevented these avenues of research from being pursued here. Yet we believe that the data presented here will be helpful in advancing the field of heritage language research for under-researched languages, such as Bosnian.

Lastly, in the current sample, only the participants from Austria reported attending Bosnian classes either in school or at the University. Austria has a well-documented availability of the so-called “mother language classes” (*Muttersprachlicher Unterricht)* at both the lower and upper levels of secondary schooling (Carnevale et al., 2007). The access to minority language instruction is not as widely available in public schools in Germany. This is apparent through anecdotal evidence reported in the media, as well as official research performed by the information platform Mediendienst Integration (Dribbusch, 2020; Mediendienst Integration, 2020; Voßkühler, 2021). BCMS is offered as a language course in public schools in only 5 out of 16 German states (Hamburg, Hessen, Rheinland-Pfalz, Sachsen-Anhalt, and Schleswig-Holstein). The lack of BCMS minority language support by German public schools is also evident in the information provided by the German participants in this study. The research into the effects of HL input and use on the development of the HL is therefore valuable, as it can ultimately help influence governmental policies on the availability of education in heritage languages. This greatly determines access to HL input during the crucial school years of child heritage speakers.

## Data availability statement

The raw data supporting the conclusions of this article will be made available by the authors, without undue reservation.

## Ethics statement

The studies involving human participants were reviewed and approved by Deutsche Gesellschaft für Sprachwissenschaft (DGfS). The participants provided their written informed consent to participate in this study.

## Author contributions

IJ, NG, and AP designed the study. IJ collected and analyzed the data. NG and her colleagues developed the narrative assessment, which was published in 2019. IJ and AP translated and adapted the narrative assessment into Bosnian. NG and AP wrote the article together with IJ. All authors contributed to the article and approved the submitted version.

## Footnotes

1 Here we do not refer to adult L2 learners: we assume that heritage grammars are different from L2 grammars in crucial ways, i.e., AoO is early rather than late and mode of instruction is naturalistic, not formal. See studies that report qualitative differences in the underlying linguistic knowledge of these populations for more information, e.g., Van Osch et al., 2018.

2 For the purpose of the discussions below, the relationship of Bosnian with Croatian, Montenegrin and Serbian must be noted. These languages until recently comprised a single language, Serbo-Croatian, which was the official language of Yugoslavia. However as Alexander (2006) remarks, it was always a “pluricentric” (p. xviii) language, which recognized several standard idioms. Following the dissolution of Yugoslavia, these distinct standard idioms came to officially form four separate languages: Bosnian, Croatian, Montenegrin, and Serbian (often investigated jointly under the umbrella of BCMS studies). Relevant findings from any of these languages will be included in our literature review.

3 Within these three main classes of declension, there are many sub-declensions based on the number of syllables and different phonological conditions which will not be discussed here (Alexander, 2006).

4 In addition to the locative case, some prepositions denoting location assign instrumental: e.g., “nad gradom” (above the city). We shall therefore refer to these as ‘location or instrumental,’ even when giving examples of prepositions assigning locative case only, as these seem more common in the sources we consulted.

5 Findings from the Raecke (2006) corpus will not be discussed here, as they primarily discuss the use of clitics in Croatian heritage speakers.

6 In the existential sentences with the verb ‘ima,’ singular noun phrases are assigned nominative case, but plural nouns are assigned genitive, see e.g., Hartmann and Milicevic (2009) for discussion.

7 As suggested to us by Boban Arsenijević, it is also possible that animacy could play some role here: the difference between accusative and nominative forms is observed only for animate, but not inanimate nouns belonging to class 1 declension. If errors are more frequent in this class than in others, such pattern can be interpreted as due to the lack of representation of the differential object marking triggered by animacy. We hope to explore this line of interpretation in future research, when more data is available.
